# The Response of Variant Histology Bladder Cancer to Intravesical Immunotherapy Compared to Conventional Cancer

**DOI:** 10.3389/fonc.2016.00043

**Published:** 2016-03-15

**Authors:** Ofer N. Gofrit, Vladimir Yutkin, Amos Shapiro, Galina Pizov, Kevin C. Zorn, Guy Hidas, Ilan Gielchinsky, Mordechai Duvdevani, Ezekiel H. Landau, Dov Pode

**Affiliations:** ^1^Department of Urology, Hadassah Hebrew University Medical Center, Jerusalem, Israel; ^2^Department of Pathology, Hadassah Hebrew University Medical Center, Jerusalem, Israel; ^3^Section of Urology, Department of Surgery, Montreal, QC, Canada

**Keywords:** urothelial carcinoma, high-grade, variants, immunotherapy, prognosis

## Abstract

**Background:**

High-grade urothelial carcinomas (UCs) often show foci of variant differentiation. There is limited information in the literature about the response of these variant urothelial tumors to immunotherapy with bacillus Calmette–Guerin (BCG). We compared the response, to treatment with BCG, of UC containing glandular, squamous, nested, and micropapillary types of differentiation to response of conventional non-muscle invasive high-grade UC.

**Methods:**

A total of 100 patients were diagnosed with variant histology urothelial cancer between June 1995 and December 2013. Forty-one patients with Ta or T1, confirmed by second look biopsies, received immunotherapy with BCG. Fourteen patients in this group were diagnosed with micropapillary differentiation, 13 patients with squamous differentiation, 9 patients with glandular differentiation, and 7 patients with nested variants. The control group included 140 patients with conventional high-grade UC. Both groups have been treated and followed similarly.

**Findings:**

Patients with variant tumors had similar clinical features to patients with conventional disease, including age, male to female ratio, stage, the presence of Tis, and median follow-up. Patients with variant tumors had a significantly worse prognosis compared to patients with conventional high-grade UC, including 5-year recurrence-free survival (63.5 Vs. 71.5%, *p* = 0.05), 5-year progression (≥T2)-free survival (60 Vs. 82.5%, *p* = 0.002), 5-year disease-specific survival (73 Vs. 92.5%, *p* = 0.0004), and overall survival (66 Vs. 89.5%, 0.05).

**Interpretation:**

A patient with variant bladder cancer treated with intravesical immunotherapy has a 27% chance of dying from this disease within 5 years compared to 7.5% chance for a patient with conventional high-grade UC.

## Introduction

The tendency of the urothelium to undergo various types of metaplasia is well-known. Foci of differentiation toward different epithelial types are frequently encountered in urothelial tumors. Histological variant urothelial carcinoma (UC) diagnosed in up to 20% of the transurethral tumor excisional biopsies and squamous differentiation is the most common type (1). Other frequently diagnosed variant UC included glandular, micropapillary, and the nested variants (2). These tumors often show clinically aggressive behavior with muscle invasion and extravesical extension (3–5). Variant tumors are frequently associated with poor prognosis (1, 2, 6). Some authors suggest that variant tumors are more aggressive compared to conventional tumors (6), although many authors maintain that the prognosis after radical cystectomy is similar in both groups.

When a patient with variant histology UC presents with muscle invasive tumor, the decision to perform radical surgery is relatively easy. When there is no detrusor invasion there is a dilemma. There are only a few reports on response of patients with variant tumors to immunotherapy with bacillus Calmette–Guerin (BCG) (7). The micropapillary pattern is an exception with several studies addressing this point in the literature, however, with inconsistent results. Kamat et al. treated 27 patients with micropapillary tumors with BCG (8). Only 19% were free of tumor after 30 months of follow-up. As such, they concluded that BCG is not effective against UC with micropapillary differentiation and suggested that radical cystectomy is the treatment for all these patients. Willis et al. arrived at a similar conclusion (9). In their study, the prognosis of patients with T1 micropapillary tumors treated with cystectomy was compared to patients treated with immunotherapy. The 5-year disease-specific survival was 100% for 26 patients who underwent immediate cystectomy compared to only 60% for the patients receiving primary intravesical immunotherapy (*p* = 0.006). Spaliviero et al., on the other hand, using similar methodology, did not find a difference in disease-specific mortality between patients treated with early cystectomy and those given primary intravesical immunotherapy.

Unfortunately, adequate information is not available on other tumor variants. In a small study, a combined series of tumor variants encouraging results for immunotherapy were reported (7). Patients with variant tumors had higher progression rate but similar disease-specific survival in comparison to patients with conventional high-grade tumors treated with immunotherapy.

In the current study, an extended group of patients with non-muscle invasive variant UCs was compared to a group of patients with conventional UC treated with intravesical immunotherapy. Both groups were treated and followed using the same protocol.

## Materials and Methods

### Patient Population

The study is based on review of the hospital database that holds information on 1210 consecutive patients who underwent transurethral resection of bladder urothelial tumors between June 1995 and December 2013. Pathological staging was performed according to the TNM system and histological grading was performed according to the ISUP/WHO classification by a single uropathologist (Galina Pizov). Patients with sarcomatoid or small cell carcinoma were not included in the study due to their poor prognosis and unique features. The study was approved by the IRB (number 207-31.10.08).

### Treatment Protocol

Patients with high-grade UC without muscularis propria invasion were subjected to the “second look” biopsies and were considered suitable for treatment with BCG if there was no invasion into the muscularis propria. The immunotherapy protocol included an induction course of six weekly intravesical instillations of OncoTICE BCG in 50 cc of normal saline, initiated 10–20 days following the second surgery. Maintenance therapy included two to three instillations every 3 months for 1 year with following procedure every 6 months for additional 2 years. Radical cystectomy was offered to patients with disease progression to stage T2 or tumors resistant to immunotherapy or recurrent high-grade, T1 or Tis.

Follow-up protocol included cystoscopy and cytology every 3 months for 2 years and every 6 months for another 3 years with a liberal policy of biopsies from any suspicious lesion. Upper tract surveillance (intravenous pyelography and more recently CT urography) was performed on initial diagnosis and then annually for 3 years.

### Statistical Analysis

The outcome of patients with variant tumors receiving BCG immunotherapy was compared to the outcome of patients with conventional high-grade UC treated with BCG during the same time period. Continuous variables were compared using *t*-test and categorical variables with the Fisher’s exact test. Survival analysis was done using the Kaplan–Meier and log-rank methods. A *p*-value <0.05 was considered significant and all tests were two-tailed. Statistical analysis was done using the JMP software (SAS Cary, NC, USA).

## Results

A total of 100 patients with variant bladder tumors were registered. These included 36 patients with micropapillary differentiation, 23 with squamous, 19 with glandular differentiation, and 22 with nested variant UC. There were no patients with lymphoepithelial or plasmacytoid variants. In 10 patients, more than one type of differentiation was noted.

Forty-one patients with non-muscle invasive disease confirmed by second look biopsies were treated with intravesical BCG. All other patients were referred for cystectomy. All patients had high-grade tumors. The clinical features of patients treated with BCG are presented in Table 1. The most common variant described was the micropapillary type. Patients with the four variants showed common clinical features (Table 1).

**Table 1:** **Features of the patients with various tumor variants**.

Variant	Number of patients	Mean age (SD)	% Males	Stages a/1 (%)	CIS (%)	Median follow-up (months)
Micropapillary	14	75.9 ± 8.8	93	4/10 (40)	7 (50)	32
Squamous	13	68.1 ± 12.6	92	0/13 (0)	6 (46)	53
Nested	7	68 ± 12.7	71	2/5 (40)	2 (29)	110
Glandular	9	75.2 ± 5.6	89	4/5 (80)	3 (33)	46
Total^a^	41	71.9 ± 10.8	88	10/31 (24)	19 (46)	43

*^a^Two patients had more than one type*.

**Figure 1:**
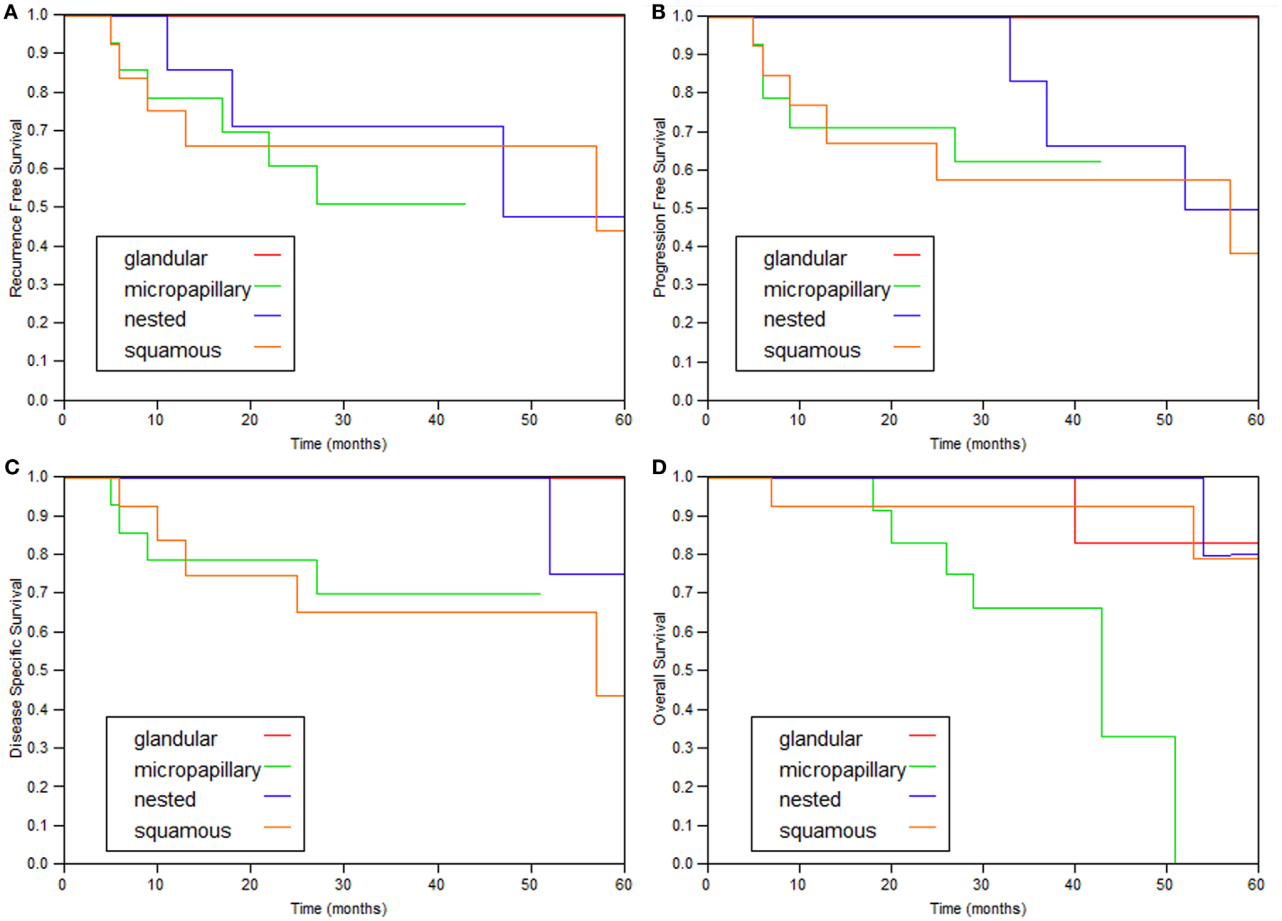
**Kaplan–Meier curves of the various variant types**. **(A)** Recurrence-free survival, **(B)** progression (≥T2)-free survival, **(C)** disease-specific survival, and **(D)** overall survival.

The 2- and 5-year survival rates of the four variants are shown in Table 2 and Figure 1 and are quite similar to each other. Patients with the glandular variant had a tendency toward better prognosis while patients with the micropapillary and squamous variants exhibited worse prognosis.

**Table 2:** **Two- and five-year survival rates according to type of variant tumor**.

Variant	Overall survival 2 years/5 years	Disease-specific survival 2 years/5 years	Progression-free survival^a^ 2 years/5 years	Recurrence-free survival 2 years/5 years
Micropapillary	75%/0%	69.8%/69.8%	69.8%/69.8%	61.1%/50.9%
Squamous	92.3%/79.1%	65.3%/43.5%	65.2%/43.5%	66.1%/44.1%
Nested	100%/0.80%	100%/75%	83.3%/50%	71.4%/23.8%
Glandular	100%/83.3%	100%/100%	100%/100%	100%/66.7%
Total^a^	92.5%/66%	85.6%/73.4%	85.6%/60%	72.3%/63.5%

*^a^To stage ≥T2*.

**Figure 2:**
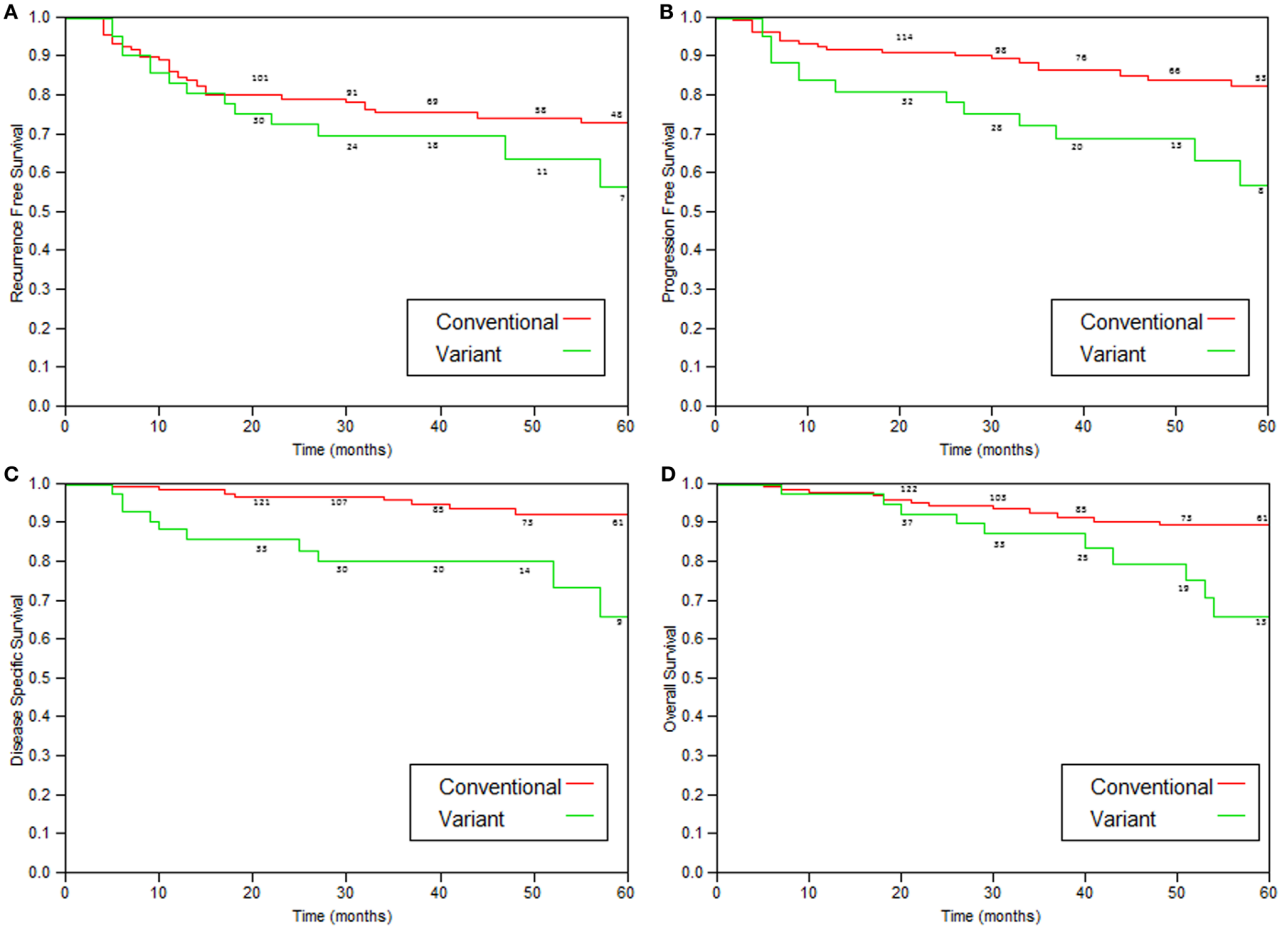
**Kaplan–Meier curves comparing variant tumors to conventional high-grade tumors treated with immunotherapy**. **(A)** Recurrence-free survival (*p* = 0.05), **(B)** progression (to stage >T2)-free survival (*p* = 0.002), **(C)** disease-specific survival (*p* = 0.0004), and **(D)** overall survival (*p* = 0.05).

Comparison of patients with variant tumors and patients with conventional high grade UC treated with BCG is presented in Table 3. Both groups were similar in age, male to female ratio, stages a/1 ratio, and the presence of Tis. Median follow-up was also similar. The recurrence-free survival, progression-free survival, disease-specific, and overall survival rates were all significantly worse for patients with variant histology tumors (Table 4; Figure 2).

**Table 3:** **Comparisons of the features of patients with conventional and variant tumors**.

	Number of patients	Mean age (SD)	% Males	Stages a/1 (%)	CIS (%)	Median follow-up (months)
Variant	41	71.9 ± 10.8	88	10/31 (24)	19 (46)	43
Conventional	140	69.9 ± 10.4	86	39/101 (27.8)	72 (51)	54
*p* Value	–	0.24	1	0.84	0.6	0.39

**Table 4:** **Comparison of the prognosis of patients with conventional and variant tumors**.

Variant	Overall survival 2 years/5 years	Disease-specific survival 2 years/5 years	Progression-free survival 2 years/5 years	Recurrence-free survival 2 years/5 years
Variant	92.5%/66%	85.6%/73.4%	85.6%/60%	72.3%/63.5%
Conventional	94.6%/89.5%	97%/92.5%	91.2%/82.5%	79.2%/71.5%
*p* Value	0.05	0.0004	0.002	0.05

## Discussion

In this study, 43 variant bladder tumors without invasion to muscularis propria founded in 41 patients and treated with intravesical BCG were studied. All four variants included in the investigation collectively demonstrate similar clinical behavior (Figure 1; Table 2).

The prognosis for patients with UC with squamous and micropapillary patterns was similar (Table 2). The 5-year disease-specific survival of patients with the micropapillary was 70%, which is comparable to the rate reported by Spaliviero et al. (75%) (10), and better than the outcomes observed by Willis et al. (60%) (9). Unfortunately, there is no reference in the literature for comparison of other variant tumors. The 5-year disease-specific survival of all the tumor variants combined was 73.4%, which is also similar to the 75% reported by Spaliviero et al., for patients with micropapillary differentiation (10).

Patients with variant tumors had similar clinical features, including age distribution, male to female ratio, stage Ta/T1 ratio, and the presence of Tis to patients with conventional high-grade tumors (Table 3).

As shown in Table 4 and Figure 2, patients with variant tumors had significantly worse prognosis compared to patients with conventional tumors in all four parameters examined (recurrence-free, progression-free, disease-specific, and overall survival rates). The fact that all the parameters were significantly worse strengthens the conclusion that these patients have poorer prognosis, especially concerning the difference in disease-specific survival. The 2- and 5-year disease-specific survival rates were 97 and 92.5% for conventional and 85.6 and 73.4% for variant tumors (*p* = 0.0004). This means that patients with variant tumors have not only a lower response rate to BCG reflected by the higher recurrence and progression rates but also lower chance for successful salvage after progression.

This study is a continuation of a previous report (7), which ­demonstrated more recurrences and shorter progression-free survivals in patients with variant tumors but similar disease-specific survival. The current study contained double number of patients and showed that disease-specific and overall survivals are also worse for patients with variant tumors.

The study is limited by the small number of patients and also by being single institutional and retrospective. The combined analysis of several variant tumors together is another limiting factor. Combining several variants is a common methodology (3, 11, 12), and as shown here, the clinical features and prognosis of the four common variants are quite similar (Tables 1 and 2).

## Conclusion

The management of patients with non-muscle invasive variant bladder tumors with intravesical immunotherapy with BCG is risky even when confirmation of diagnosis with second look biopsies and meticulous follow-up are employed. The progression rate of these patients to muscle invasive disease is high (40% at 5 years compared to 17.5% in conventional high-grade tumors). Furthermore, the chance of successful salvage, after progression is lower compared to conventional high-grade tumors. A patient with a variant tumor treated with intravesical immunotherapy has a 27% chance of dying from this disease within 5 years compared to 7.5% chance for a patient with conventional high-grade carcinomas. As such, patients with variant tumors should be advised of this adverse clinical course and considerations for cystectomy strongly recommended.

## Author Contributions

All authors listed, have made substantial, direct, and intellectual contribution to the work and approved it for publication.

## Conflict of Interest Statement

The authors declare that the research was conducted in the absence of any commercial or financial relationships that could be construed as a potential conflict of interest.
